# Impact of Information Technology on the Therapy of Type-1 Diabetes: A Case Study of Children and Adolescents in Germany

**DOI:** 10.3390/jpm4020200

**Published:** 2014-04-16

**Authors:** Rolf-Dietrich Berndt, Claude Takenga, Petra Preik, Sebastian Kuehn, Luise Berndt, Herbert Mayer, Alexander Kaps, Ralf Schiel

**Affiliations:** 1 Infokom GmbH, Neubrandenburg 17034, Germany; E-Mails: rberndt@infokom.de (R.-D.B.); ppreik@infokom.de (P.P.); skuehn@infokom.de (S.K.); lberndt@infokom.de (L.B.); 2 Department for Health Care Sciences, University of Applied Sciences, Rheine 48431, Germany; E-Mails: he.mayer@mhrheine.de (H.M.); r.schiel@medigreif-inselklinikum.de (R.S.); 3 Department of Diabetes and Metabolic Diseases, MEDIGREIF-Inselklinik Heringsdorf GmbH, Heringsdorf 17424, Germany; E-Mail: alexander_kaps@yahoo.de

**Keywords:** mHealth, diabetes management, telemedicine, app, information technology, telematics platform

## Abstract

Being able to manage and adjust insulin doses is a key part of managing type-1 diabetes. Children and adolescents with type-1 diabetes mellitus often have serious difficulties with this dosage adjustment. Therefore, this paper aims to investigate the impact of using novel mobile, web and communication technologies in assisting their therapy and treatment. A trial was conducted in the north-eastern part of Germany to evaluate the impact of the “Mobil Diab”, a mobile diabetes management system, on the clinical outcome. 68 subjects aged between 8 and 18 years, divided randomly into control and intervention groups, were included into the study. Metrics such as changes in the quality of metabolic control, changes in psychological parameters, usability and acceptance of the technology were used for evaluation purpose. Metabolic control was mainly assessed by the mean HbAlc. Analysis showed a good acceptance of the proposed system. An overall improvement in mean levels of HbA1c was observed, however further studies will be conducted to prove evidence of the weight and BMI improvements. Moreover, initial indications of positive impact on the improvement in psychological parameters were presumed based on the result of the conducted study. The system appeared to be an efficient and time saving tool in diabetes management.

## 1. Introduction

Innovations in Information Technology (IT) have improved the quality of services for people with long-term conditions such as diabetes [1,2,3,4,5,6,7,8]. The effectiveness and benefits of applying new web, mobile and communication technologies in managing diabetes have been investigated in a number of studies [9,10,11,12,13,14,15,16,17,18,19,20]. The main therapy goals for patients with chronic diseases such as Diabetes mellitus type-1 and type-2 include among others: an optimal therapy management, flexibility and independence in daily life, thus providing a better life quality and treatment satisfaction [21,22]. Optimizing the glycemic control in type-1 diabetes is important in order to minimize the risk of complications [23]. Children and adolescents with type-1 diabetes mellitus often have serious difficulties with adjustment of the insulin dose. This not only results in the impairment of reaction capabilities, decreasing of the life and treatment qualities, but also leads to acute complications of metabolic control, such as the occurrence of hypoglycemia or hyperosmolar coma, ketoacidosis [22,24].

Following the guidelines of the German Diabetes Association, the European Association for Study of Diabetes and of the American Diabetes Association, a good diabetes control can be achieved through a simple and individual therapy tailored to patients’ conditions. Moreover, theoretical and practical skills for a successful diabetes self-management and exercise should be provided to patients. Specific difficulties arise however, in case of patients with relatively complex insulin therapy regimes, for which the calculations of insulin dose are not easy. This is the case in intensified conventional insulin therapy with insulin injections (ICT—intensified conventional insulin therapy) or insulin pumps (CSII—continuous subcutaneous insulin infusion). The insulin dose must be calculated in accordance to the current blood glucose value, the planned amount of carbohydrate to be taken in the next meal, and taking into account the insulin bolus before and after the upcoming physical activity.

Another element which helps achieve a good diabetes control is the medical-psychological rehabilitation. Achieving an optimal treatment and quality of life during the rehabilitation period is not an easy process [25], especially for children, adolescents and ambulatory patients with type-1 diabetes mellitus and having a complex therapy adjustment.

Health care systems are stressed by growing costs caused by chronic illnesses [26]. Diabetes costs in Germany cover more than 10% of the national health insurance expenditures [27]. In order to remedy the situation, new technologies and telemedical applications are getting more and more important in the medical care. These new technologies can help optimize medical treatment and therapy during patient care [28,29]. The following was stated in Editorial 2011c [30]: “For some illnesses, such as diabetes mellitus, heart disease, obesity and wound care, telemonitoring services have been well tested. The economical and medical benefits have been demonstrated through studies. Some health insurance companies have therefore begun using telemedicine in the standard care”.

E-health systems store and process very sensitive data and should have a proper security and privacy framework and mechanisms since the disclosure of health data may have severe social consequences especially for patients.

Mobile apps have enabled health institutions interact with their audiences in relevant ways than traditional channels. The next step in this evolution is securing this communication so that all types of content, especially protected health information can become part of the conversation. Security of this type cannot simply be part of the app, but rather it needs to be the cornerstone of an integrated communication platform that handles the data from beginning to end and all the way through the communication.

This paper extends the state-of-the art by the points summarized as follows: Modern information technology is increasingly used in healthcare with the goal to improve and enhance medical services and to reduce costs. In particular e-health systems like electronic health records are believed to decrease costs in healthcare (e.g., avoiding expensive double diagnoses, or repetitive drug administration) and to improve personal health management in general. This task is covered by the “Mobil Diab” system which can serve as a standalone hospital information system or can exchange information with existing hospital information systems. Investigation of the impact of using this System “Mobil Diab” in assisting the therapy and treatment of diabetes is the major focus of this paper.

This paper evaluates the impact of the IT-based application “Mobil Diab”, conceived for diabetes management, on the therapy and treatment of children and teens with type-1 diabetes based on the following metrics: changes in the quality of metabolic control, changes in psychological parameters, usability and acceptance of the technology. Previous publications [31,32] have shown a rich experience in the therapy adjustment for overweight and obese children and adolescents.

The remainder of the paper is organized as follows: Section 2 describes materials and methods used to evaluate the efficiency and benefits of applying information technology on the therapy of type-1 diabetes. The diabetes management system “Mobil Diab”, study approval, recruitment for the study, study methods and experimental settings are described in that section. Section 3 presents results obtained from the study which aimed to evaluate the impact of using the system ”Mobil Diab” on the therapy and treatment of diabetes based on such metrics as metabolic control and psychological parameters. Section 4 introduces discussions summarizing observed benefits of the system from the conducted study. A short conclusion is provided in the last Section 5.

## 2. Experimental

### 2.1. “Mobil Diab” System

“Mobil Diab” system is an innovative solution for the assistance and care of diabetes patients. It enables diabetes patients manage their self-control data around the clock using their mobile devices (Android, iPhone, iPads) and/or web-based applications. Medical care access patients’ data through a protected web portal. The system is embedded on the platform which is responsible for the following tasks: Connection of web and mobile applications, user-hierarchical model (administration, hospital, doctor and patient), access control of different user categories, secure interface to hospital information systems. The concept of the system aims at empowering patients to better manage their disease by allowing them to monitor their blood sugar trends over an extended period of time and draw some actionable conclusions.

**Figure 1:**
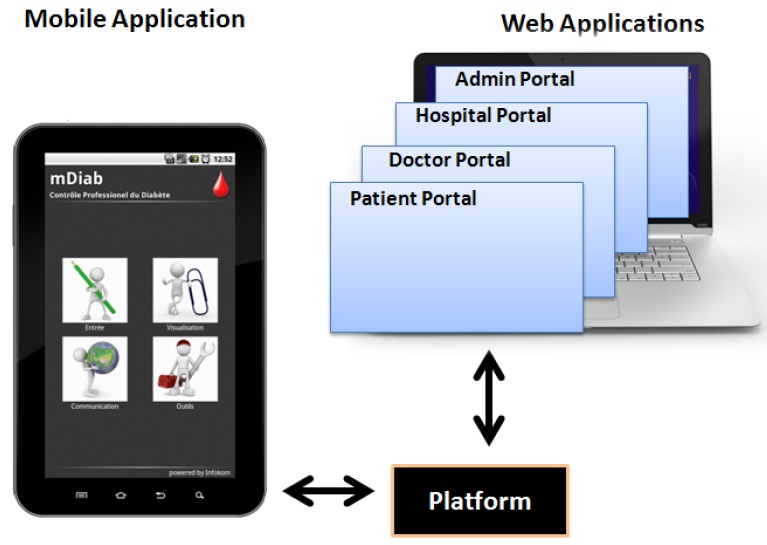
“Mobil Diab” System Components.

“Mobil Diab” system is composed by a mobile application (Android/iOS), web-based portals for different user categories and the platform as illustrated in Figure 1. It guarantees a series of benefits for patients, health care staff and public health system. The system helps to track pattern and trends in diabetes management process and also helps in taking right treatment decisions and lead to a better glucose control. It is suitable for self-care, home care, family physicians, clinics and hospitals and presents statistics that cover everything doctors and patients need. The system provides risk monitoring with automated alarm message to care provider and to trusted person. It preserves mobility through the use of Apps and web technologies and acquires a secure database platform with end-to end encryption. Data are worldwide available and the application supports multiple languages such as (English, French, German, Russian, Chinese, Spanish, Portuguese, Arabic and several others).

### 2.2. Web-Based Applications of “Mobil Diab”

The web-structure of the system is conceived in order to serve as a complete hospital management system. Four web-based portals are integrated to the system and hosted from the central platform. They are designed for four different types of users: patients, doctors, hospital administrators and system administrator. Clinicians and authorized medical staff can access patients’ data via the protected doctor portal and have a structured and understandable view of patients’ diabetes related data. Clear graphical representation of trends and statistics enable them make appropriate decisions about the therapy adjustment. This tool helps medical staff save time while providing high quality service to diabetes patients.

### 2.3. Mobile Application of “Mobil Diab”

**Figure 2:**
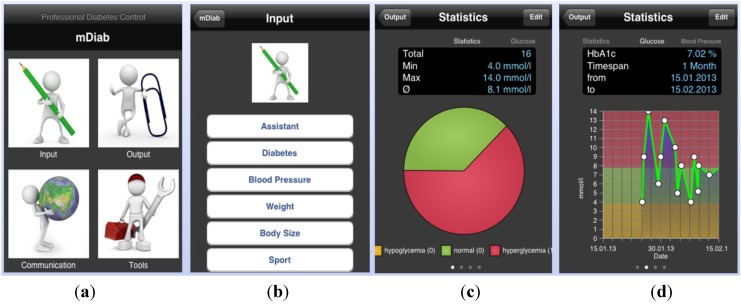
Mobil Diab Mobile Application: (**a**) Screenshots of the main screen; (**b**) input choices; (**c**,**d**) blood glucose graphs.

The mobile application (mDiab) has an intuitive user interface which is self-explanatory. You can choose the action you wish to perform as shown on the screenshot of Figure 2. Data are automatically synchronized with the central server in background to allow medical staff access them in real time.

### 2.4. Architecture of the “Mobil Diab” Platform

**Figure 3:**
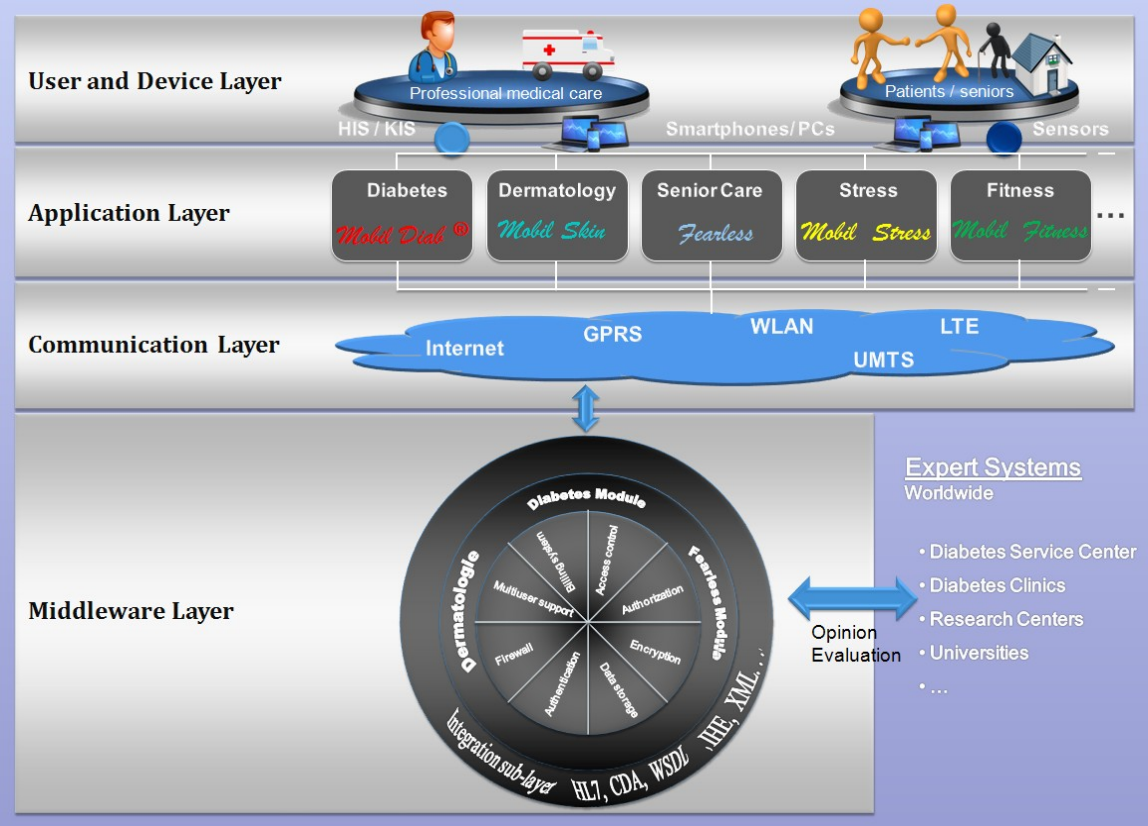
Architecture of the “Mobil Diab” Platform.

The patent-protected platform [33] illustrated in Figure 3 has been developed to help bridge the gap between health and mobility. Diverse healthcare modules have been implemented and other are planned in order to meet a complete health care service package from diabetes, dermatology, stress, fitness, long-term health conditions up to the assisted-living for senior. The platform helps consumers track health, wellness and vital information using a highly secure infrastructure. It allows consumers share information with their health care professionals and family. Moreover, interfaces to hospital information systems and practice management software are supported. The four-layer architecture of the platform enables users securely share sensitive information. Using different devices such as smartphones, computers, users can access functionalities of applications supported by the core of the platform through the communication layer.

In e-health systems, security is an imperative requirement because those systems handle very sensitive data like medical and personal data. The platform acquires features enabling:

- Authentication: methods and mechanisms which allow an entity to prove its identity to a remote end.
- Authorization: access control mechanisms and the ability of an entity to access shared resources.
- Data integrity: mechanisms which ensure that when there is an interchange of data between two peer entities, the received data and the original ones are the same, and that no intermediate alteration has occurred.
- Data confidentiality: it assures that stored or transmitted data are well protected from possible disclosure. A means used to achieve data confidentiality is through cryptographic mechanisms.
- Privacy: which can be defined as an entity’s ability to control how, when, and to what extent personal information about the entity will be communicated to third parties.
- Secure data communication and storage.
- Data availability: data can be accessed by authorized users irrespective of time and location.

Our proposed architecture addresses authentication and authorization issues, since each user is classified to a category that defines what he/she has access to. Moreover, data confidentiality is achieved through cryptographic mechanisms. Medical data and personal data are encoded and stored in separate data bases. All data are encrypted using the symmetric encryption method AES (Advanced Encryption Standard). For protecting user privacy, an implemented mechanism allows different categories of users to have different authorization levels to access protected data. Each category accesses and sees only data authorized to it through the web or mobile application. This is enabled through the integration of the authorization control mechanism in the user-hierarchy model of the system.

The platform is composed of several modules, each one supporting its group of tasks. The integration sub-layer is responsible for formatting data in a needed standard. This extends the interoperability capability with other third-party systems. Some of the supported standards include HL7, CDA, WSDL and XML. This platform was also presented in [34].

### 2.5. Ethical Approval for the Study

An ethical approval was applied to the ethics commission of the Ernst-Moritz-Arndt-University of Greifswald, Faculty of Medicine, Greifswald in Germany with the title of the study being: power analysis for the use of the diabetes management system “Mobil Diab”. The application was approved at the meeting of the Ethics Committee on the 27 July 2010 and a positive vote without further requirements was realized under the Reg.No. BB 86/10 issued by the Chairman of that Ethics Committee.

### 2.6. Inclusion/Exclusion Criteria for Subject Enrollment/Randomization

A pilot, randomized controlled trial was conducted to evaluate the impact of the “Mobil Diab” system in the treatment process of diabetes patients. Children and teens aged between 8 and 18 years, diagnosed with type-1 diabetes mellitus with disease duration of at least half a year and following the ICT or CSII therapy, were selected to take part. All included patients should acquire reading and writing skills so that they could fill the anamnesis and psychological questionnaires independently. Furthermore, all included children and teens should have no other disease apart from diabetes mellitus type-1. Those notified with other diseases during the initial examination, as well as those with weaknesses in reading and writing were not included in the test phase.

In order to estimate the minimum size of the study cohort, the statistical program G-Power 3, which is a power analysis tool, was used [35]. This revealed that a minimum size of the cohort of 68 patients must be met (power analysis: 2-sided test, effect size d = 0.7, probability error α = 0.05, power [1-β err prob] = 0.95, allocation ratio N2/N1 = 1, Result: sample size group 1: *n* = 34, sample size group 2: *n* = 34, total sample size: *n* = 68). In order to compensate an expected drop-out rate of 10%, a cohort size was raised to 75.

From diabetic patients who were successively included in the medical rehabilitation at the specialized clinic for children and adolescents MEDIGREIF Inselklinik Heringsdorf GmbH, in the period between 1 June 2010 and 31 August 31 2010, a total of 77 children and adolescents were registered and trained to participate to the study. The participation in the study was voluntary and after the written consent of the children, adolescents and their parents and a prior oral and written medical clarification. From the 77 children and teens, 4 had either another disease or did not have sufficient reading and writing skills, 5 did not agree to continue the study, and thus they were excluded. This resulted in a total number of 68 children and teens with type-1 diabetes mellitus (American Diabetes Association, 1997). All 68 patients took part until the end of the study.

For classification and evaluation purpose, the cohort was divided randomly into a control group (conventional therapy without the use of telemedicine system) and an intervention group (treatment with the use of telemedicine system “Mobil Diab”). Three groups of patients belonging to the intervention group used the mobile application of the “Mobil Diab” successively for a period of 4 weeks each. During this period, they sent their daily diabetes-related records to the central platform. These records included blood glucose measurements, nutrition and drugs information, recommended at least 3 times a day. Moreover mobility and sport-related information were recorded. This recommended frequency of supplying diabetes-related data was not met for all the days. For some few days, less record were sent to the central platform. Seven members of the medical staff involved in the study used the web-based application to access and analyze data and provide helpful feedback. From the medical staff were 2 diabetes specialists, 2 treating doctor, 2 nurses and 1 psychologist.

A comparison of the control and intervention groups in terms of anthropometric data, metabolic control parameters and diabetes-related knowledge was carried out to verify the comparability of the groups at the beginning and at the end of the study, Table 1 and Table 2. A detailed psychological diagnostic test was performed to all 68 patients. On the one hand, it should be ensured that no patient with diabetes mellitus and severe psychological problems participates in the study. The psychological test diagnostics consisted of a standardized questionnaire with different domains: Quality of life-Diabetes Quality of Life for Youth Scale (DQOLY) [36], Diabetes Family Conflict Scale [37], Overall expected self-efficacy [38], Diabetes Self-Efficacy Scale [39], Child Behavior Checklist [40], Youth Self-Report [40], Vocabulary Test [41], and Test about sequence of numbers [41]. The DQOLY scale consists of following subscales: disease impact, diabetes-related worries, treatment satisfaction, and life satisfaction. A 5-point scale was used and for life satisfaction: higher values represent better life satisfaction. For disease impact -diabetes related worries; smaller values represent better health-related life quality. In order to compare the situation at beginning and at the end, questionnaires were filled at these two stages of the study. Results of the questionnaires for the control and intervention groups are presented in Table 3.

**Table 1:** Comparison between control and intervention groups.

Parameter	Control group Mean ± SD	Intervention group Mean ± SD	*p*-value
Number (n)	34	34	/
Females (n/%)	14 (44%)	13 (38%)	0.63
Age (year)	13.2 ± 2.9	12.9 ± 2.0	0.56
Body height (m)	1.57 ± 0.15	1.62 ± 0.15	0.19
Body weight (kg) at the beginning	53.8 ± 13.9	53.7 ± 15.6	0.99
Body weight (kg) at the end	54.7 ± 14.3	53.5 ± 15.5	0.75
Body-mass Index (BMI) (kg/m^2^) at the beginning	21.8 ± 5.2	20.2 ± 3.5	0.14
Body-mass Index (BMI) (kg/m^2^) at the end	22.2 ± 5.7	20.1 ± 3.5	0.07
Diabetes duration (years)	5.3 ± 4.0	5.0 ± 3.7	0.75
Blood sugar at empty stomach during the day at the beginning (mmol/L)	8.3 ± 3.7	7.6 ± 3.2	0.38
Mean of blood glucose excursions during the first 3 days at beginning (mmol/L)	10.7 ± 3.4	8.3 ± 3.2	0.003
Mean of blood glucose excursions during the last 3 days before the end (mmol/L)	9.6 ± 3.5	8.5 ± 2.7	0.16
Patient with at least one insulin analog (n/%)	11 (32%)	9 (27%)	0.33
Number of (n) blood glucose self-tests per week	34.1 ± 8.6	36.2 ± 10.1	0.37
Strategy of the insulin therapy at the beginning (n/%):			0.60
ICT** using injections	23 (68%)	25 (74%)	
ICT** using pumps (CSII)	11 (32%)	9 (26%)	
Strategy of the insulin therapy at the end (n/%):			1.0
ICT** using injections	22 (65%)	22 (65%)	
ICT** using pumps (CSII)	12 (35%)	12 (35%)	
HbA1c (%) at the beginning	9.0 ± 2.2	8.8 ± 1.7	0.81
HbA1c (%) at the end	8.0 ± 1.3	8.1 ± 1.1	0.65
Insulin dose (I.U.) at the beginning	47.3 ± 18.3	45.5 ± 24.2	0.73
Insulin dose per kg body weight (I.U./kg body weight) at the beginning	0.89 ± 0.33	0.86 ± 0.44	0.74
Insulin dose (I.U.) at the end	46.3 ± 17.8	47.0 ± 24.4	0.89
Insulin dose per kg body weight (I.U./kg body weight) at the end	0.88 ± 0.32	0.86 ± 0.44	0.91
Number of all hypoglycemic episodes during the last month (n)*	12 (Range, 0-60)	8 (Range, 0-52)	0.24
Number of patients with at least one hypoglycemic episode during the last month (n)	33	30	0.23
Number of severe hypoglycemic episodes requiring help during the last month (n) *	0	0	0.32
Number of patients with at least one severe hypoglycemic episode requiring help during the last month (n)	1	0	0.33
Number of severe hypoglycemic episodes with loss of consciousness during the last month (n) *	1	0	0.32
Number of patients with at least one severe hypoglycemic episode with loss of consciousness during the last month (n) *	1	0	0.32
Number of ketoacidosis with hospital admission during the last month (n) *	0	0	1.0
Diabetes-related knowledge (points)	17.9 ± 4.0	17.9 ± 3.6	0.95
Blood pressure systolic (mmHg)	109.6 ± 12.2	107.9 ± 11.8	0.58
Blood pressure diastolic (mmHg)	68.6 ± 9.3	65.9 ± 9.3	0.25

Paired statistical analysis was applied for most of the cases, since our aim is in some cases to provide observations before and after an intervention on the same participant and in other cases to compare results from the same participant or statistically comparable groups using two different techniques.

### 2.7. Experimental Settings

The collection of data was performed using the computer software DPV^®^ (Diabetes-Patienten Verlaufsdokumentation, history documentation for diabetics, University of Ulm, Ulm, Germany). The statistical analysis was performed applying the statistical program SPSS (Statistical Package for the Social Sciences^®^) version 17.0 (IBM Corporation, New York, NY, USA). The descriptive data analysis was performed by analyzing frequency and specifying the mean values (Mean) with standard deviations (SD). Furthermore, in certain cases, the range (difference between the largest and smallest observed values) and the variance (arithmetic mean of squared deviations) were specified. In order to prove the reliability of the standardized questionnaire evaluating the use of the telemedicine application “Mobil Diab”, the Cronbach’s alpha (α) was calculated.

At the end of the trial each participant had to fill a questionnaire evaluating the system MobilDiab based on usability, acceptability and therapy satisfaction. For this purpose, a scale of 1.0 (=“very good”/highest score), 2.0 (=“good”), 3.0 (=“average”) to 4.0 (=“not good”/lowest score) was used. Results are summarized in Table 4; it is one score evaluating the following sub-questions:

Q1: How often could you successfully use the system?
Q2: How easy was it for you to cope with the application?
Q3: How do you evaluate the input options and design of the application?
Q4: How do you evaluate the output options and visualization possibilities?
Q5: How do you evaluate the design of the application?
Q6: Do you find the system as a tool which motivates you in the control of your diabetes?
Q7: Did feedbacks (messages, therapy) from doctors help in the diabetes management process?
Q8: Would you wish to continue using Mobil Diab^®^ for managing your diabetes?
Q9: Would you recommend the system to other users?

**Table 2:** Changes of parameters of metabolic control during the study (pairwise (intra-individual) comparison at beginning *vs.* at the end of the study), Control and intervention groups (*n* = 68 subjects with type-1 diabetes mellitus).

	Control group				Intervention group			
Parameter	At the beginning of the study Mean ± SD	At the end of the study Mean ± SD	Difference Δ Range Variance	*p*-value	At the beginning of the study Mean ± SD	At the end of the study Mean ± SD	Difference Δ Range Variance	*p*-value
Number (n)	34	34	/	/	34	34	/	/
Body weight (kg)	53.8 ± 13.9	54.7 ± 14.3	0.91 ± 2.40 10.6 5.7	0.035	53.7 ± 15.6	53.5 ± 15.5	−0.19 ± 1.59 7.3 2.5	0.501
Body-mass Index (BMI) (kg/m^2^)	21.8 ± 5.2	22.2 ± 5.7	0.40 ± 1.04 4.6 1.1	0.033	20.2 ± 3.7	20.1 ± 3.5	−0.09 ± 0.58 2.6 0.3	0.379
Mean amplitude of blood glucose excursions (mmol/L)	10.7 ± 3.4	9.6 ± 3.5	−1.12 ± 4.23 18.9 17.9	0.132	8.3 ± 3.2	8.5 ± 2.7	0.29 ± 3.03 14.9 9.2	0.580
HbA1c (%)	8.96 ± 2.23	7.99 ± 1.26	−0.98 ± 1.45 7.7 2.2	0.001	8.84±1.71	8.12±1.10	−0.72±1.48 6.0 1.2	<0.001
Insulin dose (I.U.)	47.3±18.3	46.3±17.8	−0.99±10.37 41.5 107.5	0.581	45.5±24.2	47.0 ± 24.4	1.57 ± 15.84 88.5 251.1	0.646
Insulin dose per kg body weight (I.U./kg body weight)	0.89 ± 0.33	0.88 ± 0.32	−0.02 ± 0.04 0.16 0.001	0.008	0.86 ± 0.44	0.87 ± 0.44	0.002 ± 0.03 0.18 0.001	0.566

**Table 3:** Changes in psychological parameters during the study (pairwise [intra-individual] comparison at beginning *vs.* at the end of the study), control and intervention groups (*n* = 68 subjects with type-1 diabetes mellitus).

	Control group				Intervention group			
Parameter	At the beginning of the study Mean ± SD	At the end of the study Mean ± SD	Difference Δ Range Variance	*p*-value	At the beginning of the study Mean ± SD	At the end of the study Mean ± SD	Difference Δ Range Variance	*p*-value
Number (n)	34	34	/	/	34	34	/	/
Quality of life–Impact	1.89 ± 0.35	1.97 ± 0.47	0.08 ± 0.48 2.6 0.2	0.308	2.09 ± 0.35	2.06 ± 0.38	−0.04 ± 0.38 1.8 0.1	0.572
Quality of life–Worry	1.98 ± 0.56	1.99 ± 0.62	0.01 ± 0.42 1.9 0.2	0.883	2.14 ± 0.55	2.06 ± 0.53	−0.05 ± 0.43 1.8 0.2	0.476
Quality of life–Treatment satisfaction	3.25 ± 0.68	3.46 ± 0.43	0.21 ± 0.64 3.7 0.4	0.059	3.14 ± 0.44	3.18 ± 0.78	0.04 ± 0.72 4.1 0.5	0.764
Quality of life–Life satisfaction	3.70 ± 0.74	3.80 ± 0.62	0.10 ± 0.64 3.9 0.4	0.384	3.84 ± 0.43	3.64 ± 0.83	−0.20 ± 0.98 5.1 1.0	0.241
Diabetes Self-Efficacy Scale	7.22 ± 1.64	7.65 ± 1.24	0.43 ± 1.67 9.4 2.8	0.143	7.54 ± 0.85	8.04 ± 1.22	0.49 ± 1.30 5.0 1.7	0.035

**Table 4:** Evaluation of usability and acceptance by users.

Assessment	
“very good” (1.0)	*n* = 6 (17%)
“good” (2.0)	*n* = 14 (41%)
“average” (3.0)	*n* = 5 (15%)
“not good” (4.0)	*n* = 5 (15%)
missing entries from	*n* = 4 (12%)

## 3. Results

### 3.1. Comparison Control *vs*. Intervention Group

* Value is not normally distributed, thus giving median and range, ** ICT = intensified conventional insulin therapy.

A complete comparison between the groups was performed at the beginning and at the end of the study. This comparison was made in terms of anthropometric data, metabolic control parameters and diabetes-related knowledge with aim to verify the comparability of the groups. Results are presented in Table 1. The comparative statistics for control *vs.* intervention group was done only in terms of the mean amplitude of blood glucose excursions during the first 3 days after the admission and in terms of quality of life—Impact after the admission. In terms of all other anthropometric data, parameter of metabolic control and in terms of psychological analysis, there were no differences between the two groups with the exception of a statistically significant difference in the mean blood glucose excursions during the first 3 days at the beginning. During these 3 days, measurements were taken at the clinic in the laboratory. The difference in the mean blood glucose excursions during the first 3 days could be caused by several facts. One assumption could be that, the cohort size was too small in terms of this parameter. The randomization of all the 68 patients in control and intervention group was thus successful. Both groups are statistically comparable in terms of relevant diabetes therapy and psychological parameters.

### 3.2. Changes in the Quality of Metabolic Control and Psychological Parameters

In order to assess the effect of the use of “Mobil Diab”, a paired (intra-individual) analysis of patients in both the control and intervention groups, was performed. Results at the beginning of the study were compared with those at its end, as presented in Table 2. In the control group an increase in weight and BMI was observed. Both the control and intervention cohorts demonstrated a reduction in HbA1c % during the study period.

In order to assess the effect of “Mobil Diab” on the intervention group with regards to the psychological parameters, a paired (intra-individual) analysis of the patients in the control and intervention group was carried out. Results from the beginning of the study were compared to those at its end. A significant improvement in the “diabetes self-efficacy” domain was depicted in the intervention group. This clinically means that children and adolescents with type-1 diabetes mellitus who used “Mobil Diab” have higher ability of implementing their own individual therapy demands. In the control group, no significant change in the psychological parameters was observed, Table 3.

### 3.3. Usability and Acceptance of “Mobil Diab”

Children and adolescents with type-1 diabetes mellitus who used “Mobil Diab” (intervention group) were to rate the telemedical system. For this purpose, a scale of 1.0 (=“very good”), 2.0 (=“good”), 3.0 (=“average”) to 4.0 (=“not good”) was used. The following are the results from 30/34 (88%) of the children and teens (4 (12%) of subjects did not take part in this survey).

The results of the questionnaire for the investigation of usability and acceptance of “Mobil Diab” telemedical application generally show good acceptance of the system, Table 4. Moreover, the 7 members of the medical staff who took part in the trial found the system to be helpful and time saving in analyzing patients’ data. Graphical and statistical tools integrated to the system helped them monitor some trends and draw some quick conclusions. Interaction with the patient was also easy and valuable. Benefits and some drawbacks of the system were identified by the patients and medical staff involved in the study and can be summarized as follows:

- Benefits for the patients include among others: unimpeded patient mobility, data input via smart phones and/or via web, regular self-control of diabetes-related data enables the right care to be administrated at the right time, potential to improve care process and quality of service, improvement of patients’ motivation through their involvement in the therapy process, reduced check-up frequency to doctors, use of mobile health technologies encourages diabetes patients to change their behavior/lifestyle and improve their health.
- Benefits for the health care staff involves among others the following: complete and regular data input, which is helpful for individual therapy plan, minimization of errors caused by lack of information about the disease history, improvement of the care process quality, getting specialist opinions, access to patients’ data worldwide independent from time and location, automatic alarm message in case of critical data from a patient.
- Benefits for the health system include among others the following: delay and reduce diabetes complications, minimize hospitalization rates due to diabetes complications, reduce death rates from diabetes, speed up the transition of patients from hospitals to their own homes which leads to a reduction in costs, enable the organization of health information through a structured gathering of all relevant data in one central place.
- Patients and medical staff were satisfied with the security features implemented to the system. Access to the web and mobile applications was secured by an individual password. Authorization and access to different sectors of the data was determined by the category of the user. A member of the medical staff could only coach and see patients who were allocated to him.
- A single drawback according to the users was the fact that internet connection is needed for data transfer from mobile devices to the central platform. But this was not critical, since data can be recorded several days offline and be sent when a connection is available.

## 4. Discussions

The randomization was performed successfully. The comparative statistics from the two groups (intervention *vs.* control group) of children and adolescents with type-1 diabetes mellitus depicted no clinically relevant differences with regards to the characteristics of the patients, to the parameters of the quality of metabolic control and to the results of psychological questionnaire. These two groups can be compared for the purpose of evaluating the diabetes management system “Mobil Diab”. Moreover, the psychological questionnaires used are all evaluated in different studies and the corresponding results are published. Hence, used questionnaires are very reliable and well suited for evaluation purpose of the “Mobil Diab”.

The clinical course, which means evolution/change of parameters for the assessment of the quality of metabolic control in children and adolescents with type-1 diabetes mellitus, is positive during the study for both the control and intervention groups. This is particularly evident in a significant improvement in the HbA1c values. Whereas weight and BMI showed no change in the intervention group, an increase in weight and BMI was observed in the control group. This increase is normal for children and adolescents with type-1 diabetes mellitus, but it shows a clinically adverse effect. To what extent the avoidance of this effect in the intervention group is associated with the use of “Mobil Diab”, has to be investigated in further studies. Possible would be such a correlation, which could reflect back to better documentation, tracking and control using “Mobil Diab”.

Regarding the psychological parameters that were stated at the beginning and at the end of the study, significant improvement in “diabetes self-efficacy” domain in the intervention group was observed. Clinically relevant is that children and adolescents with type-1 diabetes mellitus who used “Mobil Diab” showed better results at the end of the study in terms of “self-efficacy” and thus proving better ability to implement their own individual therapy requirements. The significant correlation in the corresponding statistical analysis between the HbA1c value and the “diabetes self-efficacy” value also shows this. Since this effect could not be proven in the control group, an association with the use of “Mobil Diab” could therefore be assumed. An exact correlation must hence be clarified in future studies.

Benefits for the patients, health care staff and health systems were listed by users involved in the study. These benefits were the same as expected and listed high flexibility, improvement in the quality of care services, time and cost saving, raise of motivation, therapy optimization, availability, speed up the transition of patients from hospitals to their own homes which leads to a reduction in costs and others as presented earlier. A single drawback according to the users was the fact that internet connection is needed for data transfer from mobile devices to the central platform. But this was not critical, since data can be recorded several days offline and be sent when a connection is available. Moreover, patients have the possibility to record measurements using whether a web-based portal or a mobile application.

## 5. Conclusions

A study evaluating the impact of using “Mobil Diab” system for diabetes management on such clinical results as psychological parameters and variables of the metabolic control has been presented in this paper. Sixty-eight Children and teens took part in the trial conducted during their medical-psychological rehabilitation period at the Diabetes Clinic in the North-Eastern region of Germany. An overall amelioration of the HbA1c values has been observed in both the control and the intervention groups; this demonstrated the positive impact of the medical-psychological rehabilitation on the average glycemic control. Moreover, results of the conducted trial demonstrated how the use of the mobile diabetes management system “Mobil Diab” obtained high patient and medical staff satisfaction scores. The use of the system resulted also in an improvement in self efficacy in the intervention group. Initial indications of its positive impact on the quality of life, weight and BMI values could be presumed based on the observed results. However, evidences for its positive impact on other variables related to the metabolic control parameters will be further investigated in our next studies over an extended period of time.

## Author Contributions

Rolf-Dietrich Berndt, Claude Takenga, Petra Preik, Sebastian Kuehn and Luise Berndt from the Infokom GmbH conceived and developed the mobile and web applications used during the study. They also implemented the whole m-health system on a secure communication platform and coordinated the whole study process. Alexander Kaps was responsible for the conducted psychological tests and their evaluations. Herbert Mayer contributed with his rich experience in statistical methods and directed tasks dealing with this subject. Ralf Schiel and his medical team coached patients during the study and supervised the whole study from the medical point of view. All authors critically reviewed and revised the manuscript, and gave final approval.
